# A Manual of Surgical Treatment

**Published:** 1900-09

**Authors:** 


**A Manual of Surgical Treatment.** By W. WATSON CHEYNE, M.B., F.R.S., and F. F. BURGHARD, M.D., M.S. Part II. Pp. xix., 382. London: Longmans, Green and Co. 1899.

The second volume of this publication maintains the high standard of excellence of the first, which was reviewed in this *Journal* (December, 1899). The subjects treated of are of great practical importance, and the interesting department of orthopædic surgery receives careful consideration. The subject of aneurysm, however, does not seem to us to receive the attention which its importance requires, though the information given is clear, and reference is made to questions of treatment still much discussed. We notice that in writing of the treatment of talipes equino-varus, Phelps's operation is favourably mentioned, and we are glad to see that the shortening of the skin is considered an objection to Lane's subcutaneous modification of it. In describing his treatment of curved tibiæ the writer maintains that splints cannot straighten the curved bones, but that if the deformity is prevented from increasing by their use, and the child is kept off its feet, the bones will grow straight of themselves. We certainly agree with the writers that as splints are used in out-patient hospital practice they probably do not mould the bones into better shape. But when we consider the way in which the ribs are gradually bent by respiratory movement, and the pelvis deformed by pressure between the femora and spine, in cases of rickets, it seems reasonable to believe that if moderate pressure can be adequately maintained on the deformed bones, they may, by this means, be gradually straightened.

Although the authors still regard Macewen's operation for genu valgum as good treatment, we are interested to see that they consider division of the tibia just below the head equally good, and that they say either method will be satisfactory in the less severe cases, but recommend a combination of both in the more severe ones.

The operation of Trendelenburg for varicose veins is referred to, though Trendelenburg's name is not mentioned in connection with it; but the authors consider that the internal saphenous vein should not be operated on unless varicose in the thigh, and that even then if other veins about the level of the knee are also varicose, portions of these should be excised as well, for they think the communication between the deep and the superficial veins above the knee is so marked that ligation of the internal saphena alone will not sufficiently reduce the backward pressure in the veins.

